# 

**Published:** 1914-12-19

**Authors:** 


### A CARDIFF MERCHANT'S WILL.

The estate of the late Mr. Richard Cory, J.P., of Oscar House, Cardiff, who died on September 20, has been provisionally estimated at about £600.000. The testator has made not a few bequests to charitable institutions, and he and his family have from time to time accorded valuable financial support to King Edward VII.'s Hospital, Cardiff.

### Personalia.

Some five members of the medical and surgical staff of the Royal United Hospital, Bath, are at present on Service. Mr. Mumford and Mr. Popham are in France; Dr. Levis is at Tidworth; Mr. Lace is medical officer to the 4th Somersets, who are stationed in Bath at Prior Park; and Dr. Waterhouse is with the 2nd Wessex Field Ambulance.

DEATH OF A HOSPITAL STEWARD.

The death has occurred at the age of fifty-eight of Mr. Joseph Watts, who was for nearly thirty-one years the steward of the Royal Hospital for Incurables, Putney Heath. Nearly three years ago Mr. Watts was operated upon by Sir Alfred Pearce Gould, and for a time his health improved; but it was found that he was not strong enough to carry on his hospital work, and he was awarded a modest pension. At the time of Mr. Watts' retirement the governors of his hospital knew to their sorrow that they were parting with one who had discharged his responsible duties over an exceptionally long period with conspicuous success. Many of the patients at Putney, who were friends of Mr. Watts for over a quarter of a century, though, strangely enough, often his senior in years, were in the habit of referring to him affectionately as "Papa"; no more simple testimony to his consideration is needed. The funeral took place at Putney Vale Cemetery, and was attended by about forty of Mr. Watts' old colleagues, as well as by two members of the board of management.

### Gifts and Bequests.

Addenbrooke's Hospital, Cambridge, has received the sum of £3,000, less duty, bequeathed by the late Mr. Gerard Cobb, of The Hermitage, Newnham, Cambridge.

The late Mr. Herbert Lee Hall has bequeathed a legacy of £500 to the London Lock Hospital. The Board have also received the following donations: E. H. M. Denny, Esq., £100; The Worshipful Company of Mercers, one hundred guineas.

### ERRATA.

HALIFAX UNION POOR-LAW HOSPITAL.

In the article on this hospital in last week's issue, page 243, first column, line 11, the reference should be to The Hospital of February 12, 1910, page 573, instead of to that of October 31 last. On page 244, first column, second paragraph, last line, the reference should be to The Hospital of October 31, 1914, page 113.

### Buildings Contemplated.

Barming.—The Kent Mental Hospitals Committee have agreed to obtain tenders for building a house for the senior medical officer at Barming Asylum. The architect is Mr. E. Newton, and the cost is estimated at £1,700.

Carnarvon.—The Town Council have passed plans for the conversion of "Bryn Seiont" into a tuberculosis hospital for the King Edward Memorial Association.

Dudley.—Plans have been adopted by the committee of the Guest Hospital for a new out-patient department and an eye dispensary.

Hanwell.—The Urban District Council have decided to apply to the Local Government Board for sanction to borrow £5,000 for an isolation hospital and furniture.

A QUESTION OF ACCOUNTS.

The following paragraph appeared in the *Times* of December 12: "At Westminster Police Court yesterday the late secretary of All Saints' Hospital, Vauxhall Bridge Road, Frederick Richard Panter, of Forel Lodge, South Hornchurch, Essex, was charged with embezzling over £500. Mr. Freke Palmer, for the prosecution, said that on investigation an accountant found that contributions from in and out patients amounting to about £300 had been stolen. Subscriptions by charitable persons had been appropriated, and in the case of a special contribution of £25 to an *x*-ray fund prisoner had forged the initials of a bank clerk to a paying-in slip. Mr. Sidney Goodman, for the prisoner, said there might have been some muddle of accounts, but he hoped to be able to clear the matter up in a satisfactory manner. Mr. Horace Smith remanded the prisoner, fixing bail in two sureties of £500 each, with notice to the police."

Rates of Subscription: Twelve months, 6/6; Six months, 4/-; Three months, 2/-, post free to any address in the United Kingdom. Abroad, Twelve months, 8/8; Six months, 5/-; Three months, 2/6, post free.—Address The Subscription Dept., "The Hospital," The Hospital Building, 28/29 Southampton Street, Strand, London, W.C.

